# Cancer-associated fibroblasts as a critical driver in tumor metastasis: The mechanisms and future perspectives

**DOI:** 10.1016/j.isci.2026.114788

**Published:** 2026-01-24

**Authors:** Lingyu Ding, Zhen Li, Jing Yue, Liqing Qiu, Zhifeng Tian, Hongfang Zhang

**Affiliations:** 1 Department of Medical Oncology, Hangzhou Cancer Hospital, Hangzhou 310002, China; 2 The Fourth Clinical Medical College, Zhejiang Chinese Medical University, Hangzhou 310053, China; 3 Hangzhou Cancer Institution, Hangzhou Cancer Hospital, Hangzhou 310002, China; 4 Department of Oncology Center, The Fifth Affiliated Hospital of Wenzhou Medical University, Lishui 323000, China; 5 Lishui Municipal Central Hospital, Lishui 323000, China

**Keywords:** Cancer, Microenvironment

## Abstract

Tumor metastasis represents a lethal event for patients due to the lack of effective treatments. Compared with primary tumors, the components of the tumor microenvironment (TME) of metastatic tumors are different. Tumor cells alone are unable to metastasize. Cancer-associated fibroblasts (CAFs), as one major component of TME, drive tumor metastasis. In metastatic lesions, the proportion of CAFs is significantly higher and is spatially close to tumor cells with high metastatic potential. CAFs themselves are resistant to chemoradiotherapy and have strong invasive ability based on their major role in degrading the extracellular matrix (ECM). Furthermore, CAFs determined the organs to which tumor cells metastasize. By interaction with tumor cells, CAFs were activated, transdifferentiated, and in turn enhanced the invasive ability of tumor cells. Tumor cells also promoted the infiltration of CAFs in tumor tissues, allowing them to establish a pre-metastatic microenvironment. In this review, we have deeply analyzed the association of CAFs and tumor metastasis from the perspectives of exosomes, metabolic reprogramming, suppression of anti-tumor immunity, and epigenetic modification. We also discussed the future perspectives of CAFs-based anti-metastasis strategies. This information may deepen our understanding of CAFs-initiated tumor metastasis and shed novel insight into the development of anti-metastasis therapies.

## Graphical abstract

**Figure:**
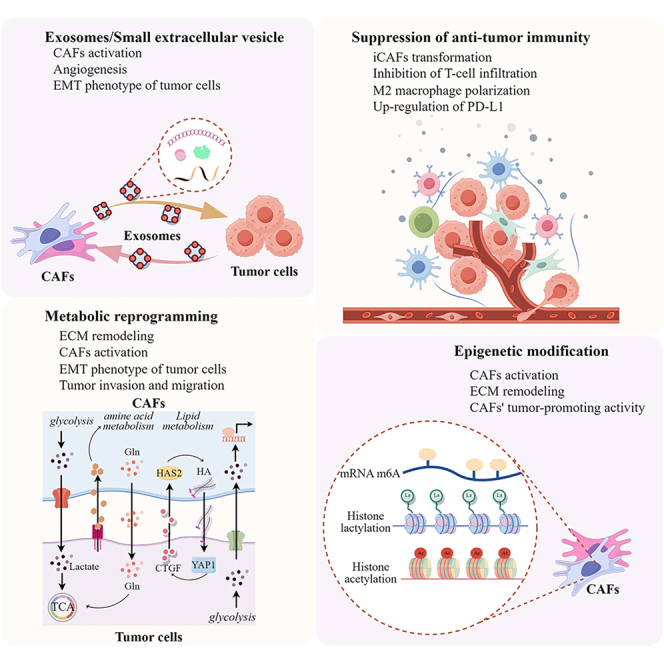

## Introduction

It is widely accepted that tumor metastasis is a lethal event for patients.^1^^,^^2^ As reported, approximately 70% of patients with tumor were diagnosed at advanced stages due to distant metastasis. And these patients have few opportunities of being cured. Compared with primary tumors, metastatic tumors show obvious resistance to the original therapeutic schedule, resulting in their rapid growth.^3^^,^^4^ Tumor metastasis is very complex and experiences multi-steps.^5^ Tumor cells need to acquire enough ability to degrade extracellular matrix (ECM), invade into and out of blood vessels, and grow into metastatic colonies in other organs. The microenvironment of metastatic tumors was demonstrated to be different from that of primary tumors, implying that tumor metastasis is closely associated with their microenvironment.^6^^,^^7^^,^^8^ Factually, tumor cells are not self-controlled during metastasis, but severely rely on stromal cells in the microenvironment, such as cancer-associated fibroblasts (CAFs). CAFs, as one major component of the tumor microenvironment (TME), support tumor initiation and progression via multiple mechanisms.^9^^,^^10^ In comparison with tumor cells, CAFs have more survival advantages following chemoradiotherapy since they have strong DNA repair and antioxidant ability.^11^ So, they may support those surviving tumor cells metastasizing after chemoradiotherapy.

Several studies have confirmed that the proportion of CAFs in the TME of metastatic tumors was significantly higher than that in primary tumors.^12^^,^^13^^,^^14^ More importantly, CAFs lead a collective invasion of tumor cells with epithelial phenotype by remodeling the ECM.^15^ CAFs generate a track through the matrix through which tumor cells invade behind fibroblasts. Tumor cells themselves have insufficient ability to achieve a collective invasion due to a lack of both force-mediated and protease-mediated matrix remodeling. In CAFs, the matrix remodeling pathways are significantly activated, while they are not essential for invasive tumor cells, suggesting that CAFs are the leading cells of tumor invasion. For tumor cells, epithelial to mesenchymal transition (EMT) is a classical mechanism for them to acquire the invasive phenotype.^16^ Only tumor cells that were spatially closer to myofibroblastic CAFs (myCAFs), one major subtype of CAFs responsible for remodeling the ECM, underwent EMT due to the stimulation of myCAFs-sourced TGF-β.^17^ Furthermore, the subtypes of CAFs determine the organs to which tumor cells metastasize, highlighting the determinative roles of CAFs in tumor metastasis.^18^

During tumor metastasis, CAFs and tumor cells were reciprocally influenced, establishing a fine-tuned interaction. CAFs release a variety of signals, including exosomes, proteins, non-coding RNAs, chemokines, and cytokines, which were taken up by tumor cells, allowing them to acquire a higher invasive and metastatic ability. Meanwhile, tumor cells enabled the promotion of the activation of CAFs via several mechanisms and the transition of their subtypes, allowing the tumor-promoting activity of CAFs to be enhanced.^19^ Muyuan You et al. demonstrated that CAFs underwent distinctive lineage transition in lung cancer brain metastasis to enhance angiogenesis, trigger metabolic reprogramming, and promote the growth of tumor cells, although their total number did not change.^20^ In addition to initiating CAFs activation, tumor cells are also enabled to promote CAFs infiltration in tumor tissues, which in turn accelerates tumor metastasis. In triple-negative breast cancer (TNBC), tumor cells-sourced epithelial membrane protein 1 (EMP1) regulated the infiltration of CAFs in tumor tissues by secreting IL-6 through the NF-κB signaling pathway.^21^ EMP1 also enhanced the proliferation of CAFs, enhancing their pro-tumor activity. To potentiate distant metastasis, even tumor cells themselves were transformed into CAFs to create a pre-metastatic niche in distant organs. Sheng Yang et al. reported that colorectal cancer cells underwent EMT to transdifferentiate into CXCL1^+^ CAFs, in which the enriched pathways were associated with characteristics of cancer epithelial cells; CXCL1^+^ CAFs further differentiated into SFRP2^+^ CAFs, in which angiogenesis, EMT, and TGFβ signaling were active to drive liver metastasis.^22^ Therefore, the cooperation of tumor cells and CAFs leads to the reshaping of TME and establishes a pro-metastatic niche for tumor cells.

Furthermore, tumor metastasis relies on an immunosuppressive environment. In recent years, immunotherapy such as immune checkpoint inhibitors (ICIs) has emerged as a novel and promising anti-tumor treatment modality.^23^^,^^24^ Especially for metastatic tumors, immunotherapy has been prescribed as a first-line therapy alone or in combination with other therapeutic options.^25^^,^^26^^,^^27^ However, the prognosis of patients treated with immunotherapy is far from satisfactory. Except for tumor cells, CAFs also interacted with immune cells and established an immunosuppressive microenvironment. This allowed metastatic tumor cells to escape from immunologic surveillance and eventually form metastatic colonies. Overall, CAFs can be used to predict or monitor tumor metastasis and function as a promising anti-metastasis target. In this review, we have deeply analyzed the association of CAFs and tumor metastasis from the perspectives of exosomes/extracellular vesicles, metabolic reprogramming, suppression of anti-tumor immunity, and epigenetic modification. We also discussed the future perspectives of CAFs-based anti-metastasis strategies. These information may deepen our understanding of CAFs-initiated tumor metastasis and shed novel insight into the development of anti-metastasis therapies.

## Exosomes/small extracellular vesicles

Exosomes are an important class of intercellular messengers in which several components, such as proteins, DNA, and RNA were included.^28^^,^^29^ Due to the lipid bilayer, exosomes are relatively stable in the body fluids such as blood, saliva, and urine and are hard to degrade, facilitating their distant movement. Through exosomes-mediated cell communication, the fates of tumor cells and stromal cells were mutually influenced. Upon the receipt of cancer cells’ signals, exosomes were released by CAFs into the TME and absorbed into cancer cells, transforming them into a pre-metastatic state.^30^^,^^31^^,^^32^^,^^33^ Serum exosomal miRNAs released by CAFs initiated the EMT phenotype of colorectal cancer (CRC) cells through targeting PTEN and were highlighted as non-invasive prognostic factors of CRC progression.^32^ In breast cancer, CAFs released exosomal *miRNA-92a* into the TME, resulting in the downregulation of *G3BP2* in tumor cells.^34^ *G3BP2* is a repressor of twist translocation from the cytoplasm to the nucleus, by which the EMT of breast cancer cells was inhibited. Except for tumor cells, CAFs-derived exosomes also regulated the behaviors of other stromal cells, such as endothelial cells, which are the main contributors to angiogenesis. Angiogenesis is essential for tumor metastasis.^35^ Nafiseh Payervand et al. reported that exosomal *circ_0084043* derived from colorectal cancer-associated fibroblasts promoted endothelial cells' proliferation, migration, and angiogenesis through the modulation of the *miR-140-3p*/*HIF-1α*/*VEGF* signaling axis.^36^ Therefore, CAFs reshape the metastatic TME by acting on several types of cells.

In turn, cancer cells-derived exosomes also promoted the activation of CAFs in order to facilitate their metastasis since cancer cells alone were hard to invade and metastasize. Under hypoxia, which is a common feature of the TME, *miR-500* was highly expressed in head and neck squamous cell carcinoma (HNSCC) cells and positively associated with their invasive and migrative ability.^37^ Furthermore, HNSCC-derived *miR-500* was secreted into the TME in the form of exosomes and functioned as an activator of CAFs by orchestrating *QKI*/*AKT*/*STAT3* signaling pathway. Activated CAFs further promoted HNSCC metastasis. Oxidative stress is another common feature of metastatic tumors because of increased reactive oxygen species (ROS) production, leading to mitochondrial DNA (mtDNA) damage and cell apoptosis.^38^^,^^39^ In order to resist oxidative stress, lung cancer cells released extracellular vesicles carrying *miR-1290,* which were absorbed into normal fibroblasts and induced their conversion to CAFs. Induced CAFs transferred mtDNA to metastatic lung cancer cells, protecting them from oxidative damage and enhancing their metastatic potential.^40^ Furthermore, by secreting small extracellular vesicles, tumor cells can even transform stromal cells in the target organs they tend to metastasize to, inducing them to establish a metastatic niche. In ovarian cancer, omental metastasis was initiated by tumor cell-derived small extracellular vesicles.^41^ By carrying *miR-320a*, these small extracellular vesicles induced adipose-derived mesenchymal stem cells (ADSCs) to show a CAF-like phenotype by activating the *TGF-β* pathway. This transition fostered omental metastasis and caused a poor prognosis for patients. Together, exosomes/small extracellular vesicles are important mediators of tumor metastasis induced by CAFs and, therefore, may be used as a non-invasive method to monitor tumor metastasis.

## Metabolic reprogramming

In addition to exosomes-mediated cell communication, metabolic communication is universal in the TME. Tumor metastasis is an energy-consuming process. During this process, the metabolism of tumor cells and CAFs is reciprocally influenced, allowing them to be in an energy-rich state. By catabolism, CAFs supported anabolism of tumor cells for their growth, survival, and metastasis. Therefore, the metabolic characteristics of metastatic tumors were significantly different compared with those of non-metastatic tumors.^42^

## Glycolysis

Glycolysis, as a major way of intracellular glucose metabolism, is critically important for the metabolic communication between CAFs and tumor cells. The CAF populations in which glycolysis metabolism was active were spatially adjacent to tumor subtypes with high metastatic potential.^43^ Upon the activation of glycolysis in tumor cells, lactate, as one main metabolite of glycolysis, was secreted into the TME and promoted the activation and recruitment of CAFs, accelerating tumor metastasis. In hepatocellular carcinoma (HCC), integrin beta 4 (ITGB4) was highly expressed in tumor cells and initiated the activation of glycolysis, which caused the activation and recruitment of CAFs, potentiating tumor metastasis.^44^ In lung cancer, tumor cells-secreted lactate was also closely associated with CAFs activation.^45^ Upon the stimulation by lactate sourced from lung cancer cells, nuclear translocation of NUSAP1 occurred in CAFs, facilitating the binding of the transcription complex JUNB-FRA1-FRA2 to the promoter of *DESMIN*. Increased *DESMIN* transcription was crucial for the activation of CAFs to promote the invasive phenotype of lung cancer cells. Activated CAFs highly secreted IL-8, promoting the recruitment of protumoral M2 tumor-associated macrophages into tumor tissues, thus creating an immunosuppressive TME to accelerate lung cancer progression. In addition to tumor tissues, glycolysis may also occur in adjacent histologically non-cancer lesions and cause the activation of CAFs, which then up-regulate the expressions of aggressive cancer-associated proteins such as MMP-2 and MMP-9.^46^ These changes led to high-risk epithelial cells, which may be the root of local tumor recurrence. Therefore, the detection of CAFs in the non-tumoral regions surrounding tumors in patients with cancer may potentially aid in the prevention of cancer recurrence and metastasis.

Autophagy is a lysosomal degradation process that supports nutrient recycling under stressful conditions.^47^ Several studies have demonstrated that autophagy is an important mechanism for CAFs and tumor cells to undergo metabolic reprogramming by activating glycolysis. By initiating autophagy, CAFs supported tumor initiation and progression. In turn, tumor cells induced autophagy of CAFs, leading to their activation and an increase in tumor-promoting activity. In tongue squamous cell carcinoma, PDGF-BB promoted autophagy of CAFs, which favored glycolysis, leading to sustained release of lactate into the TME. Then, tongue squamous cell carcinoma cells acquired enough energy to invade and metastasize.^48^

**Figure 1:**
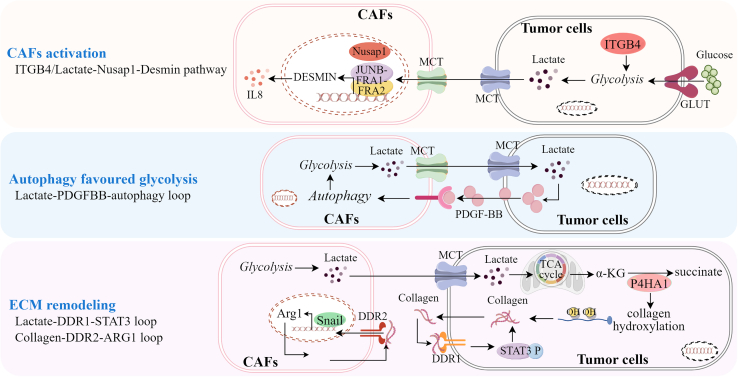
The role of glycolysis in CAFs-promoted tumor metastasis By highly expressing ITGB4, tumor cells initiated the activation of glycolysis, leading to CAFs activation and recruitment, thereby potentiating tumor metastasis. By secreting lactate, one main metabolite of glycolysis, tumor cells promoted the nuclear translocation of NUSAP1 and facilitated the binding of the transcription complex JUNB-FRA1-FRA2 to promotor of *DESMIN*, leading to CAFs activation and IL-8 secretion to recruit M2 TAMs; PDGF-BB promoted autophagy of CAFs, leading to the activation of glycolysis to release lactate; Tumor cells exploited lactate released by CAFs to produce collagens in a P4HA1-dependent manner. The collagens, in turn, bind to DDR1, trigger STAT3 activation, and lead to sustained collagen expression, supporting cell-autonomous ECM remodeling. In CAFs, DDR2, as a collagen receptor, caused the binding of snail to the promoter of *arginase*, thereby promoting collagen production and ECM remodeling.

ECM is a critical part of TME, and its remodeling is essential for tumor metastasis.^49^ CAFs were discovered as a major regulator of ECM remodeling through several mechanisms, such as initiating glycolysis. The level of lactate significantly correlated with tumor metastasis due to its involvement in regulating ECM-associated gene expressions, such as collagens.^50^^,^^51^^,^^52^ During the culture of CAFs and tumor cells, the glycolysis was significantly activated, by which CAFs controlled collagen synthesis to remodel the ECM and support tumor metastasis. In prostate cancer (PCa), CAFs produced massive amounts of lactate and released it to the TME.^53^ Lactate-exploiting PCa cells sustained an increased level of collagen hydroxylation induced by α-KG-dependent collagen prolyl-4-hydroxylase, allowing collagen to be stably synthesized. Due to the stimulation of collagen, discoidin domain receptor 1 (DDR1) was activated to promote stem-like and invasive features of PCa cells. Furthermore, the lactate-dependent loop, where binding of DDR1 to collagen triggers STAT3 activation to regulate and maintain collagen expression, supported cell-autonomous ECM remodeling to sustain metastatic behavior in prostate cancer. In ovarian cancer, discoidin domain receptor 2 (DDR2), as a fibrillar collagen receptor, was highly expressed in omental CAFs and promoted collagen production through increasing the binding of snail to the promoter of *arginase*.^54^ High stromal arginase-1 expression therefore remodeled the ECM and supported ovarian cancer cells’ colonization in the omentum, leading to poor survival of patients. In addition to tumor-filtrating CAFs, circulating CAFs (cCAFs) were present in the plasma of metastatic patients and closely associated with tumor metastasis because they were viable and enabled to secrete collagen.^55^ The graphical representation of the role of glycolysis in CAFs-promoted tumor metastasis is shown in Figure 1.

### Amino acid metabolism

Except for glycolysis, the metabolism of amine acid is also critically important for CAFs to maintain their tumor-promoting phenotype. In ovarian cancer, SLC7A1, as a transporter of amino acids, was highly expressed in CAFs and was involved in TGFβ1-induced CAFs activation by reprogramming amino acid metabolism. High expression of SLC7A1 in CAFs then promoted the invasion and migration ability of ovarian cancer cells.^56^ Glutamine (Gln), as a non-essential amino acid in the body, determines the invasive phenotype of CAFs as well as their ability to promote tumor cells’ invasion.^57^ CAFs tend to move toward the area where a high concentration of Gln is present in the TME. Gln contributes to the activation of CAFs in a TGF-β1/Snail1 signaling axis-dependent manner. Activated CAFs enhanced epithelial tumor invasion toward Gln, while tumor cells alone did not show dependence on Gln in the process of invasion. Recently, Gln was demonstrated to be a critical player during the metabolic reprogramming of CAFs and tumor cells, which governed tumor progression. In triple-negative breast cancer, a highly aggressive malignancy with dismal prognosis, estrogen-activated G protein-coupled estrogen receptor (GPER) regulated glutamine production and release by CAFs, which were then absorbed into tumor cells and utilized to promote mitochondrial activity and tumor progression.^58^ Blockage of GPER-mediated Gln metabolic reprogramming significantly inhibited TNBC progression.

### Lipid metabolism

Lipid metabolism is also an important source of energy for tumor cells and may be reprogrammed during the interaction of tumor cells and CAFs. In colorectal cancer (CRC), distant metastases, particularly liver metastasis, account for 90% of CRC-related deaths.^59^ For patients with CRC with steatotic liver, which is induced by a high-fat diet, the tumors metastasized to the liver more easily compared to those without steatotic liver. This difference implied that the metabolism of lipid may be involved in the liver metastasis of patients with CRC. Recently, Yoon Mee Yang et al. revealed that steatotic liver promoted CAF infiltration and collagen and hyaluronic acid (HA) production in the CRC metastatic niche.^60^ By highly expressing HA synthase 2 (HAS2), CAFs activated yes-associated protein (YAP) in CRC cells, which then released connective tissue growth factor to further activate CAFs for HAS2 expression. CAFs-derived HAS2 increased the infiltration of M2 macrophages, where PD-L1 was highly expressed and induced the exhaustion of CD8^+^ T cells, creating a pro-metastatic microenvironment to accelerate CRC liver metastasis. CD36, as a fatty acid transporter, was an important anti-tumor target because of its involvement in tumor proliferation, metastasis, and immune escape. Furthermore, CD36 was associated with CAFs activation by regulating lipid metabolism. In hepatocellular carcinoma, CD36 was responsible for the reprogramming of lipid metabolism in CAFs, producing higher content of lipid droplets and high expression of autophagosomes.^61^ This CD36-mediated metabolic reprogramming enabled CAFs to display tumor-promoting activity. Down-regulation of CD36 inhibited the proliferation and migration of CAFs. In addition, CD36^+^ CAFs increased the expression of PD-1 in CD8^+^ T cells, resulting in their exhaustion. Therefore, hepatocellular carcinoma cells grow in a pro-metastatic microenvironment produced by CD36^+^ CAFs.

**Figure 2:**
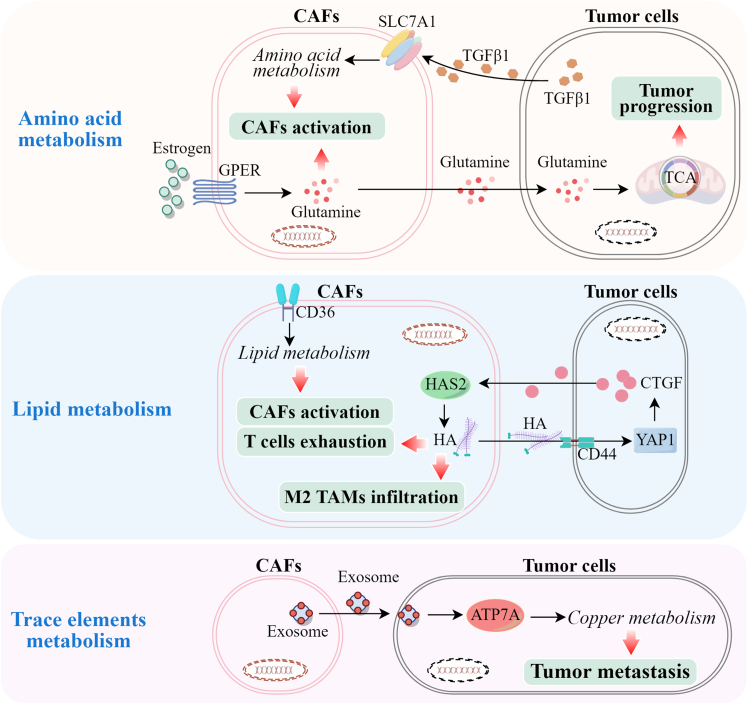
The roles of amino acid, lipid, and copper metabolism in CAFs-promoted tumor metastasis High expression of SLC7A1 in CAFs initiated their activation to promote the invasion and migration ability of tumor cells; GPER regulated glutamine production and release by CAFs, which were then absorbed into tumor cells and utilized to promote mitochondrial activity and tumor progression; By highly expressing HAS2, which regulated HA production, CAFs activated YAP through CD44 in tumor cells and promoted CTGF release to further activate CAFs for HAS2 expression. This signaling loop ultimately enhanced the infiltration of M2 macrophages and T cell exhaustion; CD36 reprogrammed lipid metabolism to induce CAFs activation, resulting in T cell exhaustion. Exosomal *miR-148b-3p* secreted by CAFs induced up-regulation of ATP7A, a transporter of copper, resulting in increased cuproptosis and tumor metastasis.

In addition to glucose, amino acid and lipids, the metabolism of trace elements such as copper also has a significant influence on tumor metastasis. The disorders of copper metabolism may induce cuproptosis, a type of cell death, which is different from apoptosis, necrotizing apoptosis, iron death, and pyroptosis. Shuaiyuan Zhang et al. found that the reduction of exosomal *miR-148b-3p* secreted by CAFs induced up-regulation of ATP7A, a transporter of copper, which increased cuproptosis and tumor metastasis in oral squamous cell carcinomas.^62^ The graphical representation of the roles of amino acid, lipid, and copper metabolism in CAFs-promoted tumor metastasis is shown in Figure 2.

## Suppression of anti-tumor immunity

Even though energy supply is satisfied, the tumor cells which succeeded in metastasis must escape from immune surveillance, the functions of immune cells have an important influence on tumor metastasis. Several studies have demonstrated that tumor metastasis relied on the establishment of an immunosuppressive microenvironment.^63^^,^^64^^,^^65^^,^^66^ CAFs have the ability to establish an immunosuppressive microenvironment via several mechanisms to help metastatic tumor cells escape from immune surveillance.

## Suppression of cytotoxic T cell infiltration

CD8^+^ T cells are the major anti-tumor immune cells, and their activation and infiltration into tumor tissues are directly associated with tumor response to immune therapy. Therefore, a high percentage of CD8^+^ T cells in tumor tissues was positively associated with the patients’ prognosis. As described above, CAFs are major sources of producing ECM-related proteins, which function as barriers to immune cell infiltration into tumor tissues. So, CAF/ECM density was inversely correlated with T cell infiltration. CAFs' destruction reshaped the tumor stroma to favor T cell infiltration and tumor inhibition.^67^ In cervical cancer, the CAF density has a negative correlation with CD8^+^ T cells in the tumor and a positive correlation with CD8^+^ T cells in the stroma.^68^ And there was a negative correlation between CAFs density and CD8^+^ T cells T:S (Tumor:Stroma) ratio, suggesting that CAFs repressed the infiltration of CD8^+^ T cells into cervical cancer tissues. Furthermore, either CAFs density or the CD8^+^ T cells T:S ratio was significantly associated with lymph node metastases in cervical cancer. However, only the combination of CAFs and CD8^+^ T cells T:S ratio has better predictive performance compared with a single index.

## Transformation of inflammatory cancer-associated fibroblasts

CAFs are heterogeneous and have distinct functions. Inflammatory CAFs (iCAFs), as a major CAF subtype, contributed significantly to the creation of an immunosuppressive tumor microenvironment. To facilitate metastasis, tumor cells are enabled to transform the phenotype of CAFs into inflammatory subtypes.^13^ By secreting inflammatory factors, iCAFs regulated the proliferation, activation, function, and infiltration of immune cells. In cutaneous T cell lymphoma, which is characterized by the presence of clonal malignant T cells, CAFs showed an active role in the TME, and the occurrence of iCAFs represented the advanced stage of this disease [91]. Through mutual positive feedback of the crosstalk between tumor cells and CAFs during the CTCL progression, tumor cells promoted iCAF generation, and the iCAFs, in turn, improved the invasiveness and metastasis of the malignant T cells.

Pancreatic ductal adenocarcinoma (PDAC) is a highly aggressive malignancy. As reported, approximately 50% of patients with PDAC were diagnosed at the metastatic stage. Even though the patients were treated with radical surgery, there are still 20% of patients who experienced tumor relapse due to hepatic metastasis within 2 years. Inflammation is a key factor in inducing an immunosuppressive tumor microenvironment. In PDAC, CALB2 caused the transformation of pancreatic stellate cells (PSCs) into iCAFs, which enhanced PDAC metastasis by creating an inflammatory TME, leading to tumor immunosuppression.^69^ Furthermore, by activating the IL6-STAT3 inflammatory signaling pathway, CAFs induced the up-regulation of CALB2 in PDAC cells, which then initiated the activation of Ca2^+^-CXCL14 inflammatory axis to facilitate PDAC metastatic outgrowth and immunosuppression.

## Induction of tumor-associated macrophages

TAMs are a major type of immune cells and can be polarized from antitumor M1 phage to protumor M2 phage under stressful conditions. Several studies have demonstrated that the increase of TAMs infiltration in tumor tissues was closely associated with tumor migration and invasion. In order to metastasize, tumor cells are able to activate CAFs and promote the transformation and recruitment of TAMs. In head and neck squamous cell carcinoma, a novel subtype of CAFs characterized by high expression of Secreted Frizzled-Related Protein2 (SFRP2_CAFs) was discovered to be spatially closer to TAMs in metastatic lesions.^70^ SFRP2_CAFs originating from smooth muscle cells displayed unique characteristics resembling both mCAFs and iCAFs. By secreting CCL2, a well-known chemokine involved in TAMs recruitment, SFRP2_CAFs recruited TAMs, where the interaction between MIF-CD74 ligands and receptors present on CAFs and TAMs was enhanced, resulting in increased invasive and metastatic abilities of tumor cells. In esophageal squamous cell carcinoma metastasis, chromatin accessibility promoted the differentiation of TAMs and CAFs.^71^ In colorectal cancer, CAFs secreted Collagen triple helix repeat containing 1 (CTHRC1), a 30 KDa protein, to promote macrophage’s M2 polarization and infiltration into tumor tissues, resulting in colorectal cancer progression.^72^ A retrospective clinical study also demonstrated that the percentage of TAMs was positively associated with CAFs in colorectal cancer tissues.^73^ Furthermore, the percentage of M2-type macrophages as well as CAFs was higher in patients with colorectal cancer with distant metastasis than that in patients without metastasis, suggesting that CAFs could promote the chemotactic ability of macrophages, potentiating their infiltration into tumor tissues to establish an immunosuppressive microenvironment. In pancreatic cancer, CAFs also facilitated M2 macrophage polarization through secreting exosomal PTGS2, which activated the NOD1 pathway, leading to enhanced tumor metastasis.^74^ In soft tissue sarcoma, Michinobu Umakoshi et al. demonstrated that the combination of CAFs and tumor-associated macrophages (TAMs) has significant prognostic values.^75^ They evaluated the expression of intratumoral CAFs as well as marginal CAFs using the markers, including FAP, CD10, and podoplanin. Their study identified intratumoral FAP/CD10 and marginal FAP/podoplanin/CD163-positive macrophage scores as independent prognostic factors for metastasis-free survival.

## Increase in PD-L1 expression

PD-L1 is known as an immunosuppressive molecule when expressed in tumor cells or stromal cells because its binding with the receptor PD-1 causes the dysfunction of cytotoxic T cells. In several human cancers, CAFs were demonstrated to up-regulate the expression of PD-L1 in tumor cells, thus creating an immunosuppressive TME to accelerate tumor progression.^76^^,^^77^^,^^78^ In patients with locally advanced nasopharyngeal carcinoma, CXCL1^+^FAP^+^CAFs were discovered as an independent prognostic factor of distant metastasis.^79^ The patients with CXCL1^+^FAP^+^ CAFs showed higher expression of PD-L1 in tumor tissues, suggesting the close association of tumor metastasis and immunosuppression. However, this study did not reveal how CXCL1^+^FAP^+^CAFs up-regulated PD-L1 expression in tumor tissues. In addition to tumor cells, high expression of PD-L1 in stromal cells is also an indicator of poor prognosis. In head and neck neoplasms, tumor cells recruited and transformed bone marrow mesenchymal stem cells (BM-MSCs) into CAFs.^80^ Meanwhile, BM-MSCs contributed to HNSCC invasiveness by increasing PD-L1 expression in both HNSCC cells and BM-MSCs, causing a reciprocal support in favoring tumor aggressiveness. Therefore, PD-L1 may be highly expressed in several types of cells due to the complex interaction of TME components.

**Figure 3:**
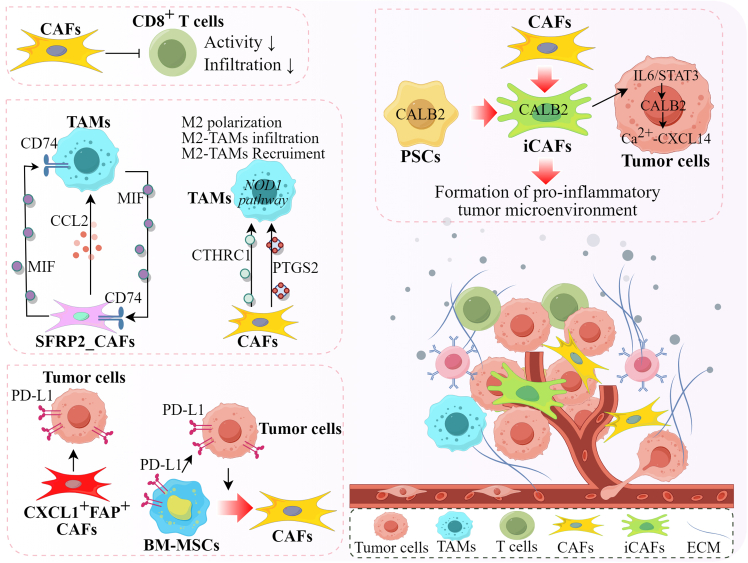
CAFs established an immunosuppressive environment to promote tumor metastasis CAFs inhibited the activity and infiltration of CD8^+^ T cells by producing ECM-related proteins; CALB2 caused the transformation of PSCs into iCAFs. By activating the IL6-STAT3 pathway, iCAFs up-regulated CALB2 in PDAC cells, which initiated the activation of Ca2^+^-CXCL14 inflammatory axis to facilitate metastasis; By secreting CCL2, SFRP2_CAFs recruited TAMs where the interaction between MIF-CD74 ligands-receptors present on CAFs and TAMs was enhanced, resulting in tumor metastasis; CAFs secreted CTHRC1 to promote macrophage’s M2 polarization and infiltration into tumor tissues; CAFs also facilitated M2 macrophage polarization through secreting exosomal PTGS2 through the NOD1 pathway; CXCL1^+^FAP^+^CAFs up-regulated PD-L1 in tumor tissues; Tumor cells recruited and transformed BM-MSCs into CAFs. BM-MSCs increased PD-L1 expression in both tumor cells and BM-MSCs.

Above all, CAFs play a determinative role in the establishment of an immunosuppressive microenvironment. The combination of CAFs and immune cells can be used as an effective tool to assess the risk of tumor metastasis. The graphical representation of CAFs-induced immune suppression in tumor metastasis is shown in Figure 3.

## Epigenetic modification

As described above, the crosstalk of tumor cells and CAFs is very complex and diverse. Epigenetic alteration is another important mechanism of CAFs to promote tumor metastasis. Epigenetic dysregulation, such as DNA methylation and histone modifications, is an important hallmark of human cancer due to its involvement in regulating the expression of key genes involved in tumor progression.^81^ Through epigenetic alteration, CAFs acquired the ability to promote tumor metastasis. TGF-β1 is known as a classical inducer of CAFs activation.^82^^,^^83^^,^^84^ In osteosarcoma, tumor cells-secreted TGF-β1 promoted the activation of mesenchymal stem cells (MSCs) into CAFs to potentiate tumor progression.^85^ Mechanical studies revealed that the overexpression of methyltransferase-like 3 (METTL3) in osteosarcoma cells enabled the stabilization of TGF-β1 signaling by m6A modification, therefore inducing the constitutive activation of MSCs into CAFs. As described above, the components of metastatic microenvironment differ from those in primary tumors. Furthermore, the gene signature of metastatic tumors and stromal cells is also different from those in primary lesions, possibly due to epigenetic modifications. Therefore, metastatic lesions may not be responsive to routine treatment regimens that were prescribed to primary lesions.

Pediatric liver cancers often develop lung metastasis. Ruhi Gulati et al. revealed that the metastatic microenvironments of pediatric liver cancers contain CAFs and neuron-like cells, which initiated cancer spread from the liver to the lungs.^86^ They found that cisplatin combined with SAHA, an inhibitor of histone deacetylase (HDAC), enabled the elimination of metastatic cells in pediatric liver cancers, while cisplatin alone did not achieve this effect. Further studies revealed that the *HDAC1*-*Sp5* pathway was activated in metastatic CAFs, which rendered them sensitive to the combination treatment of cisplatin and SAHA. In colorectal cancer, the subtype of CAFs highly expressing sulfatase 1 (SULF1) promoted tumor metastasis by remodeling the ECM through enhancing the release of VEGFA from heparan sulfate proteoglycan. When the tumors were treated with the HDAC inhibitor chidamide, the expression of SULF1 in CAFs was significantly decreased, and the progression of tumors was simultaneously inhibited. In addition to acetylation, histone lactylation also regulates the tumor-promoting activity of CAFs. Bo Gao et al. reported that MFAP5^+^ CAF promoted the formation of extramural venous invasion (EMVI), an independent risk factor for distant metastasis in advanced gastric cancer by regulating lactylation modification, which enhanced EMT pathways, although the involved mechanisms were not entirely clear.^87^ Therefore, epigenetic therapy may be a promising strategy of targeting CAFs to repress tumor metastasis.

## Discussion

Increasing evidences have demonstrated that the populations of CAFs are significantly heterogeneous.^88^ As reported, there are mainly three subtypes of CAFs, including myofibroblasts (mCAFs), inflammatory CAFs (iCAFs), and antigen-presenting CAFs (apCAFs).^89^ mCAFs and iCAFs were responsible for the ECM remodeling and creation of an immunosuppressive TME to promote tumor progression, respectively. The functions of apCAFs have remained inconsistent because they were demonstrated to enhance or inhibit antitumor immunity.^90^^,^^91^ In addition, there are also some newly discovered subtypes of CAFs demonstrating anti-tumor activity.^92^ Therefore, it is essential to discover the heterogeneity among CAF populations and define the subtypes of initiating tumor metastasis through single-cell RNA sequencing (scRNA-seq). Based on the molecular characteristics of CAFs subtypes, the risk prediction model of tumor metastasis can be established.^93^^,^^94^^,^^95^^,^^96^

Since CAFs drive tumor metastasis, the detection of CAFs distribution in the body may be earlier to recognize tumor metastasis. FAP, as a key biomarker of CAFs, has been often used to target CAFs to repress tumor metastasis by the establishment of nano-drug delivery systems (NDDSs).^97^ NDDS have the advantages of precise and effective delivery of multi-target drugs and visualized functions to trace the distribution of drugs.^98^ By establishing FAP-targeted and chemotherapeutic agents-encapsulated NDDS, both tumors and CAFs can be attacked to realize a dual anti-tumor metastasis effect.^99^ Furthermore, by targeting FAP expressed on CAFs, we can also identify high risk of areas for tumor metastasis by tracing CAFs. In hepatocellular carcinoma (HCC), a novel near-infrared (NIR) fluorescence imaging strategy was developed to target FAP^+^ CAFs.^100^ This technology enabled to visually provide the spatial distribution heterogeneity of CAFs by NIR fluorescence. The researchers found the targeted fluorescence was relatively enriched more in the blood flow direction and at the tumor edge, both of which were associated with tumor metastasis, confirming the high association of CAFs distribution and tumor metastasis. In the future, this promising method may be adopted in several types of tumors to realize the early detection of tumor metastasis by tracing CAFs. Although CAFs-based NDDS showed promising anti-tumor effects in preclinical studies, none of them have entered the clinical trial stage due to various difficulties and challenges.^101^ In our opinion, the main reason is that the 3D structure of tumor tissues is far more complex than what we know now; in addition, there is persistent and dynamic crosstalk between tumor cells and stromal cells. All of these greatly restricted the directional delivery of nano-drugs. On the other hand, until now, more specific biomarkers of CAFs are lacking, because the FAP-targeted nano-delivery systems may have off-target effects.^102^ Therefore, we need to understand the tumor tissues more clearly through modern molecular techniques such as single-cell spatial multi-omics to perform the integration analysis of the crosstalk between tumor cells and CAFs. Then, the design of CAFs-based nano-delivery systems may be more intelligent.

Furthermore, as described above, extracellular vesicles (EVs) such as exosomes played important roles in the crosstalk of tumor cells and CAFs to promote tumor metastasis. EVs are very stable and can facilitate long-distance intercellular communication. More importantly, highly metastatic tumor cells-derived EVs had the tropism to the specific organ or cell type in distant metastasis.^103^^,^^104^ The EVs with high expression of integrin α6β4 showed tropism to lung fibroblasts and therefore can be used as a tool by packaging to target and modulate the phenotype of CAFs in distant metastasis.^105^ Furthermore, inhibiting the secretion of exosomes by CAFs is another promising anti-tumor metastasis strategy. TAS2R9, as a G protein-coupled receptor, is highly expressed in CAFs. Peptide HTTIPKV, which specifically binds to TAS2R9, was linked to ultrasound-activated nanodroplets to target CAFs.^106^ Meanwhile, these nanodroplets contained imipramine, an inhibitor of exosomes, to block the secretion of exosomes by CAFs to inhibit tumor metastasis. Therefore, EVs have several functions in the development of anti-tumor metastasis strategies.

In addition to labeling CAFs, there are also some other ways to detect metastatic CAFs as early as possible. ECM proteins, such as collagens, which are mainly produced by CAFs, have an important influence on tumor metastasis. The detection of Type XII collagen in the serum can distinguish patients with cancer from healthy controls as a non-invasive tool.^107^ Therefore, by detecting the proteins that are secreted by metastatic CAFs into the serum, tumor metastasis may also be early discovered to realize precision therapy of patients. In addition, CAFs themselves can circulate in the blood, and several studies have demonstrated that circulatory CAFs (cCAFs) can be detected in the blood of patients with cancer. Furthermore, cCAFs were positively associated with circulating tumor cells (CTCs), which were the root of tumor metastasis. The positivity of cCAF in tumor patients’ blood was significantly associated with lymph node metastasis and lower EPCAM levels on CTCs, suggesting that cCAFs may protect cCTCs, leading to tumor progression. In patients with metastatic tumors, the number of cCAFs was significantly associated with the overall survival of patients.^108^ Therefore, the detection of cCAFs in the blood of patients may be another attractive approach for monitoring tumor metastasis. However, the development of a more sensitive and standard method to detect cCAFs in the blood of patients with cancer is urgent.

Overall, targeting CAFs is an attractive method of inhibiting tumor metastasis due to their intimate association. However, this aim relies on a deeper understanding of the cell communication of CAFs and tumor cells as well as the pathological role of CAFs in tumor metastasis.

## Acknowledgments

This work was financially supported by the 10.13039/501100001809National Natural Science Foundation of China (No. 82472935), the Zhejiang Medicine and Health Science and Technology Project (No. WKJ-ZJ-2549, No. 2024KY204, No. 2023KY193, and No. 2025KY1114) and The Construction Fund of Key Medical Disciplines of Hangzhou.

## Author contributions

L.D. and Z.L. searched the literature and wrote the article. J.Y. and L.Q. prepared the figures. H.Z. and Z.T. revised and approved the article.

## Declaration of interests

The authors declare no conflict of interest.
